# Antibodies for the Coat Protein of Cotton Leafroll Dwarf Virus Detect *Commelina* sp. as an Intermediary Host for Cotton Blue Disease

**DOI:** 10.3389/fpls.2022.814119

**Published:** 2022-07-13

**Authors:** Lucia Vieira Hoffmann, Amanda Alves Branquinho, Paulo Augusto Vianna Barroso, Maite F. S. Vaslin

**Affiliations:** ^1^Brazilian Agricultural Research Corporation (EMBRAPA), Brasília, Brazil; ^2^Biological Science Institute, Universidade Federal de Goiás, Goiânia, Brazil; ^3^Virology Department, Universidade Federal do Rio de Janeiro, Rio de Janeiro, Brazil

**Keywords:** enzyme-linked immunosorbent assay (ELISA), CLRDV, virus, intermediary host, serology

## Abstract

The cotton blue disease, caused by the cotton leafroll dwarf virus (CLRDV), leads to dwarfism, leaf rolling, and production loss in susceptible cotton varieties. To develop an enzyme-linked immunosorbent assay (ELISA) test to detect the virus in cotton and weeds, peptides based on the coat protein were used to produce polyclonal (α-GQE, α-PRN, and α-INK) and monoclonal (α-GQE, α-PRN, and α-NKF) antibodies. All six were tested as capture antibodies, and polyclonal α-GQE and the monocle onal α-NKF were labeled with the enzyme alkaline phosphatase and used as detection antibodies for a double antibody sandwich (DAS) ELISA method, in which p-nitrophenyl phosphate was added and measured by absorbance at 405 nm. The DAS-ELISA sandwich was efficient in discriminating between healthy and diseased plant extracts. The ELISA methodology detected the virus in the weeds *Commelina* sp., which was confirmed by RT-PCR. The monoclonal antibodies may be used to develop other diagnostic procedures.

## Introduction

Cotton blue disease is one of the most important diseases of cotton, caused by the cotton leafroll dwarf virus (CLRDV; Correa et al., 2005). It is transmitted by the aphid *Aphis gossypii* (Cauquil and Vaissayre, 1971; Michelotto and Busoli, 2007). Virus resistance in cotton is conferred by a single locus (Pupim et al., 2008). Single nucleotide polymorphisms (SNPs) and simple sequence repeats (SSRs) markers linked to the resistance gene *Cbd* have been identified (Fang et al., 2010). The presence of atypical symptoms has been observed in cotton fields and may be related to the variability of the virus (Silva et al., 2008). Important luteoviruses were detected in cotton in Australia (Ellis et al., 2013) and Argentina, respectively, called Cotton bunchy top virus, CBTV, and Cotton leafroll bushy virus (CLRBV). CLRDV has been reported in Brazil (Correa et al., 2005), Argentina (Distéfano et al., 2010), India (Mukherjee et al., 2012), Thailand (Sharman et al., 2015), Timor-Leste (Ray et al., 2016), Uzbekistan (Moukahel et al., 2021), and Sudan (Kumari et al., 2020). Furthermore, it has been reported in various states in North America, first in Alabama (Avelar et al., 2019), and later in Mississippi (Aboughanem-Sabanadzovic et al., 2019), Georgia (Tabassum et al., 2019), Texas (Alabi et al., 2020), Kansas (Ali and Mokhtari, 2020), and Florida (Iriarte et al., 2020).

The detection of the virus by reverse-transcriptase (RT)-PCR is laborious and expensive. Until now, no diagnostic serological methods are available and previous attempts to detect the virus by serology using general antisera against luteovirids failed (Takimoto, 2003). Even more sensitive than RT-PCR assays, ELISA represents an important tool as it is cheaper, easier to use in basic labs, and less susceptible to host inhibitors. Weeds have been recently reported as CLRDV’s secondary hosts in Georgia, detecting the virus by RT-PCR (Sedhain et al., 2021). The objective of this work was to develop a rapid diagnostic test for the presence of the virus, and its presence in the weed *Commelina* sp. was detected.

## Materials and Methods

Three peptides (Table 1) were chosen from the sequence of the CLRDV coat protein using the Emini plot (Emini et al., 1985) to combine antigenicity, flexibility, and hydrophilicity. To prepare the polyclonal antisera, each of the peptides was conjugated with keyhole limpet hemocyanin (KLH). Modeling, synthesis, and conjugation were conducted at the Federal University of São Paulo. Peptides were injected twice at 1-week intervals into rabbits (five rabbits per synthetic peptide, two doses of 2.5 mg of peptide for each rabbit) to obtain polyclonal antiserum. Initial bleeds were tested against the antigen to decide when to take further blood extraction. The titer measurement was taken with the same peptides conjugated to BSA (bovine albumin).

**TABLE 1:** Polyclonal antiserum titers according to peptide sequence.

Designation	Amino acid sequence	Number of amino acids	Titers from rabbit serum
α-GQE	GQEWHDTSEDQFR	13	1600*
α-PRN	PRNTQRRRRRRRGGRNRTG	19	500
α-INK	INKFGITKNGRKQFA	15	500

*The same titer was obtained for this antiserum in two productions.

The monoclonal antibodies were obtained for the same GQE and PRN peptides described in Table 1, plus the α-NKF 14 amino acid peptide, NKFGITKNGRKQFA. They were produced by Rheabiotech Co., Sao Paulo, SP. www.rheabiotech.com.br. Three different groups of mice were individually inoculated with each peptide (α-GQEWHDTSEDQFR; α-PRNTQRRRRRRRGGRNRTG and α-INKFGITKNGRKQFA) using Complete Freund Adjuvant at first dose, and Incomplete Freund Adjuvant at second and third doses (0, 14, and 28 days). After a blood test on day 35, a booster dose in phosphate-buffered saline (PBS) was administrated and after 7 days, the splenocytes were fused with SP2/Ag0 myeloma cells to obtain different hybridomas that secrete specific monoclonal antibodies. The monoclonality was achieved by limit dilution, and the specificity of these monoclonal antibodies was evaluated by ELISA.

After some initial tests with labeled goat anti-rabbit antiserum, the polyclonal α-GQE and the monoclonal α-NKF were conjugated to the enzyme alkaline phosphatase. Cotton plants grown in a greenhouse, covered with an aphid-proof screen, were used for DNA extraction and PCR to verify the presence of the SSR marker DC20027 and assess the presence of the resistance gene, expected in the CLRDV-resistant cotton varieties (Delta Opal and BRS 293), and absent on those reported as susceptible (Fibermax 966, CNPA GO33, and CNPA 809) (Pupim et al., 2008; Fang et al., 2010; Menezes et al., 2014). For cotton plant inoculation, *A. gossypii*, fed on diseased cotton plants for 3–5 days, was transferred to the resistant or susceptible plants with a brush. Six viruliferous aphids were placed on each plant, for 48 h, and then, were manually eliminated.

*Commelina* sp. plants colonized by *Aphis gossypii*, with a slight abaxial leaf curling when compared to the neighbor plants of the same species, were collected in the field in Santo Antônio de Goiás and Campo Grande, Brazil, and transplanted into pots in a greenhouse.

The 96 polystyrene microtiter plates with “U” bottom were coated by the different monoclonal or polyclonal antibodies diluted in sodium carbonate buffer (pH 9.6). The incubation with the capture antibodies was for 4 h (preliminary procedures) or 2 h (adjusted procedures) at 37^°^C. Different antiserum dilutions on sodium carbonate buffer (pH 9.6) of 1:100, 1:150, 1:180, 1:200, and 1:250 (v/v) were tested during the preliminary procedures, and antisera diluted at 1:180 were used thereafter. At the end of each incubation period, the ELISA plate was washed four times with PBST (10-mM phosphate buffer pH 7.4 plus 0.5% Tween 20). The same microtiter plate wells were submitted to a second incubation period with BSA diluted at 3% in saline phosphate buffer (w/v), 200 μl/well, for 2 h at 37^°^C, and subsequently washed four times with PBST. Then, 100 μl/well of plant extract was added. The plant extract was obtained from the maceration of 60 mg of fresh weight of plant material (petiole, leaf, and stem), plus 350 μl of PBS (10-mM phosphate buffer pH 7.4), and centrifuged at 8,000 *g* for 30 min at 4^°^C, after which the supernatant was collected. The incubation with plant material was 16 h at room temperature (preliminary procedures) or 2 h (adjusted procedures), at 37^°^C. The plate was washed again four times with PBST and incubated for 2 h, 37^°^C, with 100 μl/well of the antiserum conjugated to alkaline phosphatase enzyme (GQE as a polyclonal antibody or NKF as monoclonal antibody), diluted 1:100 on sodium carbonate buffer (pH 9.6). After a final set of PBST washes, the substrate p-nitrophenyl phosphate diluted in diethanolamine buffer (1 mg/ml) was added. The absorbance at 405 nm was measured 30 min later. At least one extraction buffer control not containing plant extract was made in each plate.

Weed double antibody sandwich (DAS)-ELISA was performed with triplicates of each sample to obtain the average of three A405 values. Results were validated by CLRDV CP amplification by nested RT-PCR following Silva et al. (2008) procedures.

Average DAS-ELISA values obtained by healthy and CLRDV-infected plants were compared using the *F*-test. One-way ANOVA with the Bonferroni test was applied for comparing ELISA’s readings.

## Results

**FIGURE 1:**
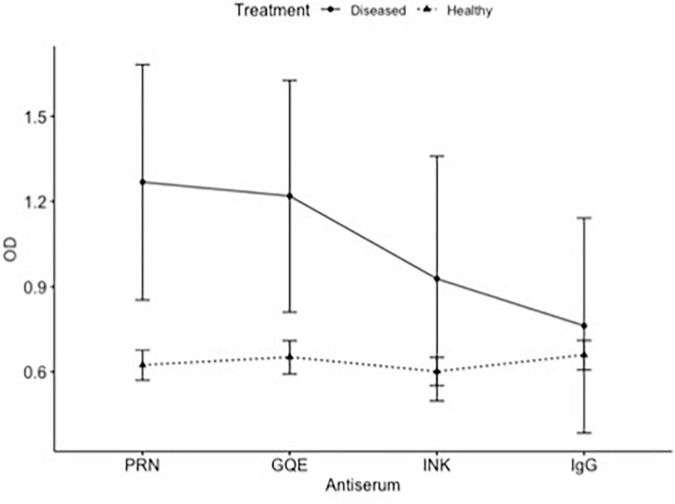
ELISA of healthy and diseased plants. Comparison of absorbance (OD) of the same plants obtained by different antiserum.

The three polyclonal antisera (PRN, GQE, and INK) were compared as capture antibodies, always using GQE conjugated to alkaline phosphatase as the detection antibodies. The comparison between the antisera analyzing leaves from the same 16 plants showed that the ELISA reads were higher for CLRDV-inoculated plants than healthy ones when PRN or INK was used as capture antibodies (Figure 1).

The PRN and INK antisera were most promising in this test and did not differ significantly from each other in a second trial. Using only the PRN and INK antisera, a new quantification test was performed at the same dilutions as the previous test. The INK antiserum showed a proportional increase between the reading and the concentration of diseased plant extract added. The antiserum PRN did not accurately detect this addition of viruses in the extract; however, it differentiated the healthy plant from the diseased plant. There was no statistical difference between the two antisera (*p* = 0.798).

The healthy extract differed significantly from the 50% diseased extract (*p* = 0.04) and the 100% diseased extract (*p* = 0.005) when using the INK antiserum to cover the plate, in the 1: 100 dilution, in increasing proportions of the diseased plant extract. The mixtures containing 0, 25, 50, 75, and 100% of diseased plant extract could be distinguished by ELISA.

The DAS-ELISA with monoclonal antibodies differentiated plants with and without symptoms. The three antiserums were tested to coat the plate, and the second antibody was NKF conjugated to alkaline phosphatase. The concentration of viral coat protein measured by monoclonal antibodies ELISA, 9 days after inoculation, was greater for the susceptible variety FM966 (1.77) than for the resistant variety BRS293 (0.67), when measured at leaves higher than the inoculated (*p* < 0.01 according to the *F*-test, average values for eight plants of each variety).

**FIGURE 2:**
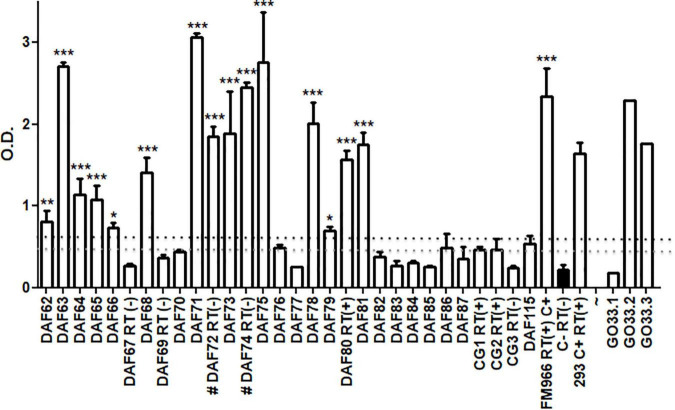
ELISA assay for 30 independent *Commelina* plants (DAF62-87, DAF 115, and CG1-3) collected from field in two distinct localities of Brazil. Averages and SD of OD values from three technical replicates of each plant are showing. GO33.1-3 represents healthy cotton plants where virus was transmitted from aphids collected from *Commelina* positive and negative plants. The OD values of each plant were compared with 3 healthy cotton plants used as negative control (in black). Statistical analyses were performed using one-way ANOVA with the Bonferroni test with *p* < 0.5. The black dashed line shows a value corresponding to 3 times the average of negative control and the gray dashed line shows 2.5 times values. These dashed lines are representing the ELISA cut-off. RT – plants assayed by RT-PCR. C+ - FM966 and 293 CLRDV infected plants. *p < 0.05, **p < 0.01, and ***p < 0.001.

*Commelina* sp. weeds collected in the field, with or without leaf curling, presented great variability in ELISA readings (polyclonal antibodies) (Figure 2).

The symptoms were too faint to discriminate between the presence of aphids only or also viruses, thus it was not possible to correlate symptoms and absorbance readings. High readings differed significantly from others that are considered healthy, suggesting that a great amount of viral coat protein may be presented in some infected plants. Among 30 *Commelina* plants assayed by ELISA, 15 showed high OD levels and were considered positive for CLRDV, taking into account a cut-off of 3 times OD values from a cotton-healthy control. Considering a 2.5 times cut-off, 21 plants may be considered positive. This indicated that half of the collected plants had the virus and could be secondary hosts of this pathogen. Thirty *Commelina* sp. plants were tested by nested RT-PCR and the presence of coat protein RNA was observed in three of the *Commelina* sp. plants positive for ELISA, DAF 80, CG1, and CG2 (Figure 2). For two ELISA-positive plants, a negative RT-PCR result was obtained probably due to the low RNA quality of these samples.

Aphids collected from *Commelina* sp. plants were transferred to healthy cotton plants in a greenhouse. After 30 days, plants were assayed by ELISA. In Figure 2, representative results are shown. Aphids collected from CLRDV-positive plants were able to transmit CLRDV to two out of three healthy cotton plants.

## Discussion

The antisera produced are efficient in ELISA tests for virus detection and can be used for diagnosis and quantification of the coat protein of CLRDV. The antiserum identifies that the virus is present even in cotton plants without symptoms and secondary hosts (weeds) and can continue being used for epidemiology research and physiology of the host reaction. However, further validation of the ELISA protocols described should include testing of healthy plants of each weed species to exclude any cross-reaction with endogenous proteins. In the case of the *Commelina* sp. that we have identified as an alternative host of CLRDV, it would increase the confidence in this finding if healthy plants were grown from seed before testing. It would also be useful to identify this host to species level by either morphological characters of mature plants or by sequencing plant barcoding genes.

The identification of blue disease has been often done through symptoms, but variations of the disease can arise, which makes the use of symptoms an unreliable diagnostic method. Cotton production in Brazil and other countries cover a wide geographical area and is often not located close to advanced virology laboratories, so a more robust method like ELISA can more easily be performed closer to production areas and, in doing so, control measures can be undertaken.

Different *Commelina* species can be found as invasive plants in several cotton-growing regions (Culpepper et al., 2004; Freitas et al., 2018). Tolerance to glyphosate has been reported (Freitas et al., 2018), therefore, its incidence tends to increase and be present during the entire cycle in fields formed by GM cotton cultivars tolerant to this herbicide. *Commelina* sp. are alternative hosts of the CLRDV aphid vector, *Aphis gossypii*, therefore, in regions where CLRDV is present, this weed may be an important reservoir of the virus. Reducing the presence of *Commelina* and the aphid vector within the field may help to decrease CLRDV pressure in susceptible cotton varieties.

Other weeds can be scrutinized as potential secondary hosts. Other methods for virus detection and quantification are more laborious, such as reverse transcription (Correa et al., 2005; Silva et al., 2008) or quantification by Real Time-PCR/Sybr-green (Chomič et al., 2011). Intermediary hosts for CLRDV were identified in the United States (Sedhain et al., 2021). Our report of *Commelina* sp. is the first report of an intermediary host of CLRDV in Brazil. Even though the protocol described here could be further validated, the antisera produced were able to be used in CLRDV serological assays. The use of diagnostic methods including the ELISA presented here will enable a better understanding of virus epidemiology and assist in developing sustainable disease management strategies.

## Data Availability Statement

The raw data supporting the conclusions of this article will be made available by the authors, without undue reservation.

## Ethics Statement

Ethical review and approval was not required for the animal study because Monoclonal antibodies have been produced by Rheabiotech (https://www.rheabiotech.com.br/) and polyclonal antibodies by Celula B (https://www.ufrgs.br/celulab, ethical committee: https://www.ufrgs.br/ceua/). Brazilian law has been followed. Our institution did not conduct any animal experiments.

## Author Contributions

AB performed the ELISA tests and RT-PCR. LVH and PB conducted polyclonal and monoclonal antiserum designs and supervised all the experimental procedures. MFSV designed and supervised RT-PCR for CLRDV. LVH wrote the manuscript. LVH and MFSV reviewed the manuscript. All authors contributed to the article and approved the submitted version.

## Conflict of Interest

The authors declare that the research was conducted in the absence of any commercial or financial relationships that could be construed as a potential conflict of interest.

## Publisher’s Note

All claims expressed in this article are solely those of the authors and do not necessarily represent those of their affiliated organizations, or those of the publisher, the editors and the reviewers. Any product that may be evaluated in this article, or claim that may be made by its manufacturer, is not guaranteed or endorsed by the publisher.
